# New Insights on the Effects of Methylphenidate in Attention Deficit Hyperactivity Disorder

**DOI:** 10.3389/fpsyt.2020.531092

**Published:** 2020-09-30

**Authors:** Maria Bernarda Pitzianti, Simonetta Spiridigliozzi, Elisa Bartolucci, Susanna Esposito, Augusto Pasini

**Affiliations:** ^1^Division of Child Neuropsychiatry, Department of Neuroscience, University of Rome Tor Vergata, Rome, Italy; ^2^Department of Child Neuropsychiatry, USL Umbria 2, Terni, Italy; ^3^Paediatric Clinic, Pietro Barilla Children’s Hospital, Department of Medicine and Surgery, Università of Parma, Parma, Italy

**Keywords:** attention-deficit/hyperactivity disorder, dopamine transporter gene, executive functions, human endogenous retrovirus, methylphenidate

## Abstract

This narrative review describes an overview of the multiple effects of methylphenidate (MPH) in attention-deficit/hyperactivity disorder (ADHD) and its potential neurobiological targets. It addressed the following aspects: 1) MPH effects on attention and executive functions in ADHD; 2) the relation between MPH efficacy and dopamine transporter gene (DAT) polymorphism; and 3) the role of MPH as an epigenetic modulator in ADHD. Literature analysis showed that MPH, the most commonly used psychostimulant in the therapy of ADHD, acts on multiple components of the disorder. Marked improvements in attentional and executive dysfunction have been observed in children with ADHD during treatment with MPH, as well as reductions in neurological soft signs. MPH efficacy may be influenced by polymorphisms in the DAT, and better responses to treatment were associated with the 10/10 genotype. Innovative lines of research have suggested that ADHD etiopathogenesis and its neuropsychological phenotypes also depend on the expression levels of human endogenous retrovirus (HERV). In particular, several studies have revealed that ADHD is associated with HERV-H over-expression and that MPH administration results in decreased expression levels of this retroviral family and a reduction in the main symptoms of the disorder. In conclusion, there is a confirmed role for MPH as an elective drug in the therapy of ADHD alone or in association with behavioral therapy. Its effectiveness can vary based on DAT polymorphisms and can act as a modulator of HERV-H gene expression, pointing to targets for a precision medicine approach.

## Introduction

Attention-deficit/hyperactivity disorder (ADHD) represents one of the most common neurodevelopmental disorders with onset in early childhood, high heritability and documented brain abnormalities (1). ADHD is diagnosed on the basis of persistent inattention, hyperactivity, and impulsivity lasting more than 6 months and interfering with subject function and development (2). According to a recent meta-analysis, ADHD prevalence in pediatric population is 5.9%–7.1% and in adults is 5% (3). Over a lifetime, ADHD symptoms cause global dysfunctions with socio-economic impact with a significant effect in various areas, including education, employment, and quality of life (4).

Since the 1990s, neuroimaging studies (5, 6) and genetic research (7, 8) have revealed that dopaminergic and frontostriatal system dysfunction represent neurobiological substrates of ADHD. Moreover, the dopamine (DA) transporter gene (SLC6A3) encodes the DAT protein, which controls the DA concentration at the synaptic level through its reuptake and influences the susceptibility to ADHD as well as response variability to methylphenidate (MPH) in subjects with ADHD (9, 10). MPH is the most commonly prescribed psychostimulant in the pharmacological therapy of patients with ADHD (11) and its efficacy is complemented by behavioral strategies (12, 13).

A variable number of tandem repeats (VNTR) in the DAT 3’-untranslated region (3’UTR) has been the most investigated polymorphism of this gene. The three most common alleles are represented by the 10/10 genotype, 9/10 genotype, and 9/9 genotype, and each has been reported with differential gene expression, influencing DAT availability (14, 15). Studies in animal models, genetic research, and human neuroimaging reports have suggested an altered availability or function of DAT in ADHD (16). One study showed a positive correlation between DAT availability in the striatum and inattention in patients with ADHD compared to healthy children (17). The positive correlation between DAT and inattention could reflect lower and shorter DA signaling in subjects presenting a higher DAT concentration (17). Several studies seemed to show that non-responders to MPH among children with ADHD have a low primary striatal DAT availability, whereas patients with a better response to MPH treatment have a higher DAT concentration. Other researches have reported a better response to MPH in patients with the 10/10 genotype and/or 9/10 genotypes compared to subjects with the 9/9 genotype (18, 19), and recent evidence has shown higher striatal DAT concentrations in subjects homozygous for the 10/10 genotype (18).

This narrative review addresses the following aspects: 1) MPH impact on attention and executive functions (EFs) in ADHD; 2) the relation between MPH efficacy and DAT polymorphism; and 3) the role of MPH as an epigenetic modulator in ADHD. We decided to perform a narrative review because our aim was to focus on precision medicine approaches and available studies did not permit to reach this goal with a systematic review.

## Attentional Functions and Their Neurobiological Bases

**Figure 1:**
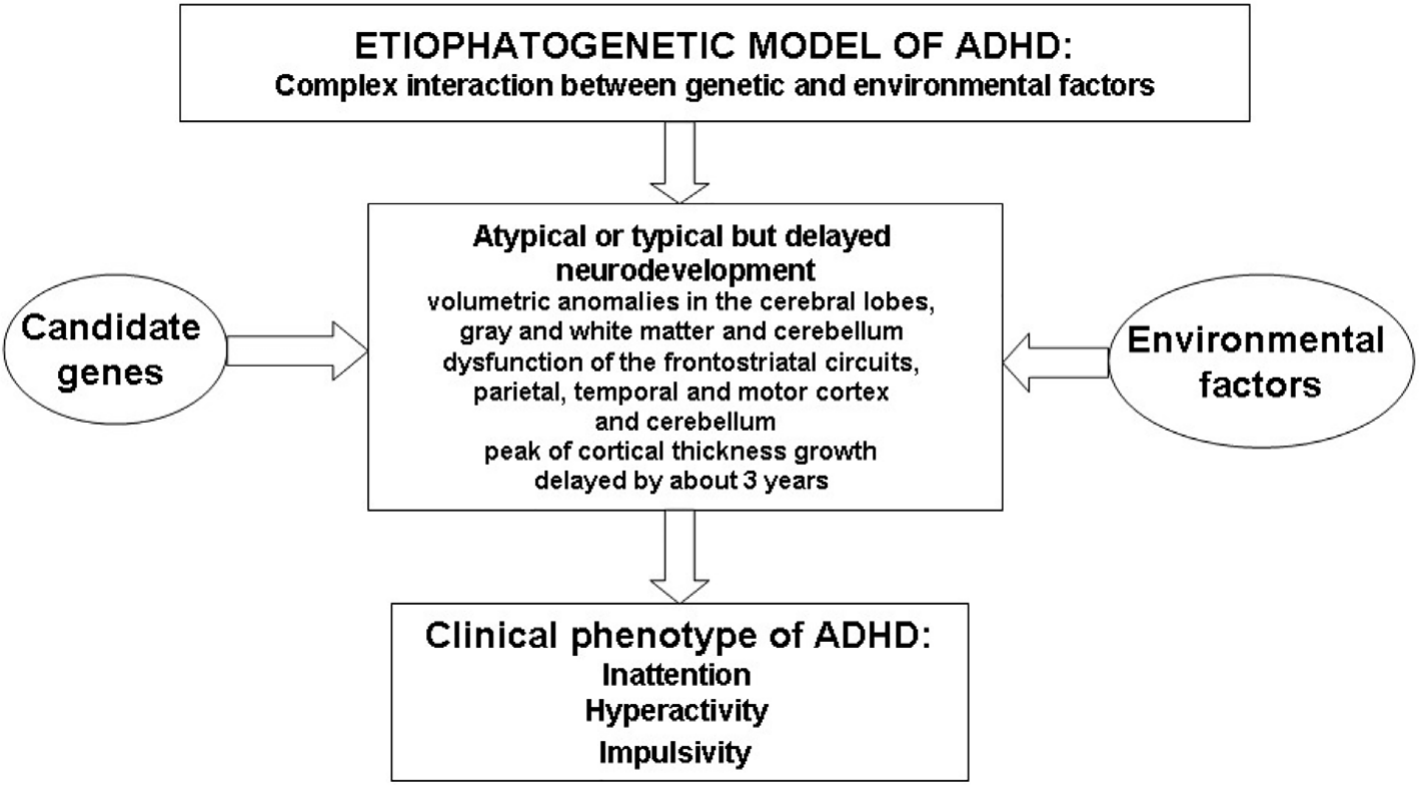
Schematic overview of etiopathogenesis of Attention Deficit Hyperactivity Disorder (ADHD).

Sustained attention is the capacity to direct attention to one or more sources of information over a relatively long and unbroken period of time; vigilance, as a type of sustained attention, is the ability to maintain attention over a prolonged period during which infrequent response-demanding events occur (20–24). Selective attention is the capacity to focus attention in the face of distracting or competing stimuli (20–22). Divided attention is needed to respond simultaneously to multiple tasks or multiple task demands (20–22). Finally, strategy/flexibility is the ability to shift the focus of attention to ensure that information from competing sources will be selectively processed (24). **Figure 1** illustrates the complex etiopathogenetic mechanisms responsible for the onset of the ADHD clinical phenotype.

Attentional functions are mediated by several cortical and subcortical networks; however, the processing of specific functions is related to specific brain regions. Alertness functions are supported by the reticular formation, dorsolateral prefrontal cortex and inferior parietal cortex (25). The reticular and intralaminar thalamic nuclei and the anterior parts of the cingulate gyrus support vigilance/sustained attention (25). The anterior cingulate gyrus, the inferior frontal cortex, in particular that of the left hemisphere, and the frontal-thalamic association connections to the reticular nucleus of the thalamus are crucial for selective attention (26). Divided attention is supported by the prefrontal cortex, in particular that of the left hemisphere, and anterior cingulate gyrus (27). Strategy/flexibility depends on the inferior parietal cortex, superior colliculus, and posterior lateral thalamus, in particular, the pulvinar and medial frontal areas, including the rostral and caudal cingulate zones and the pre-supplementary motor area (28).

## Attentional Dysfunction and Methylphenidate

Attentional dysfunction is one of the main symptoms of ADHD. Regarding attention deficits, parents and teachers say that patients with ADHD have difficulties concentrating, paying attention to details and sustaining attention for a prolonged period of time (29). Disturbances in arousal, selective attention, sustained attention, shifting, and distractibility are the most prominent deficits of attention reported in children with ADHD (30). Children with ADHD reported an impairment in measures of alertness compared to children with typical development, which appears to be due to low levels of arousal (31); moreover, they showed an impairment in measures of selective and divided attention and an increased variability in reaction times in sustained attention tasks compared to their healthy peers (32). The reaction time and its variability, considered a measure of attention (33), may be affected by symptoms commonly observed in children with ADHD, including distractibility, deficient self-regulation of motivation and/or impaired perseverance (34, 35). Evidence of increased variability in reaction time in ADHD led to the formulation of a mechanistic hypothesis of default network interference.

Sonuga-Barke and Castellanos postulated that deficient regulation of the default network by cognitive and attention networks highlights intrusions in their interplay that manifest as phasic lapses of attention or in impulsive behaviors (36). This hypothesis was supported by several studies that showed abnormalities involving the default network in subjects with ADHD (37, 38). Applying the multi-component model of attention of Van Zomeren and Brouwer (33), Tucha and colleagues reported that patients with ADHD showed deficits in attention, comprising impairments in vigilance, selective attention, focused attention, divided attention, and shifting compared to healthy peers (39, 40). Using the same multi-component model of attention, Pitzianti and colleagues found that in comparison to healthy peers, children with ADHD were seriously impaired in attentional processes, including alertness, selective attention, divided attention, and sustained attention (41). Taken together, these studies show an extensive dysfunction of cortical and subcortical networks that support attentional processes in ADHD. It is known that MPH acts by increasing the concentrations of DA and norepinephrine in the synaptic cleft by blocking their reuptake (13).

Several studies have shown that DA and norepinephrine participate in the processes of attention and inhibition (42, 43), and low levels of these two neurotransmitters cause dysfunction in the above-mentioned processes. This explains MPH efficacy in reducing inattention in children with ADHD, as well as the other main symptoms of the disorder (44, 45). Indeed, MPH has been reported to improve performance on measures of attention, memory and EFs (46, 47). Regarding MPH effectiveness, parents and teachers describe that pharmacologically treated patients improve their social interactions, oppositional and aggressive behavior, and classroom behavior (48). A meta-analysis of studies using a continuous performance test (CPT), considered a vigilance/sustained attention measure, revealed that omission errors (lack of response to target stimuli assumed to reflect inattention), and commission errors (responses to non-target stimuli assumed to reflect impulsivity) were sensitive to medication in children and adults with ADHD who received MPH (49, 50).

MPH treatment has been shown to decrease reaction time and its variability (40) and to improve tonic alertness (51), phasic alertness (18), divided attention (52), flexibility/shifting of attention (47), and aspects of selective attention such as inhibition (53) and focused attention (54) in children with ADHD. These findings were confirmed by a study on MPH efficacy, in which Tucha and colleagues (39), using the multi-component perspective of attention of Van Zomeren and Brouwer (33), evaluated the attentional performance of children with ADHD before and after MPH administration. In this study, before MPH treatment, patients with ADHD showed marked impairments in vigilance, divided attention, flexibility, and aspects of selective attention, including focused attention, inhibition, and integration of sensory information (39). Attentional dysfunction also manifested itself through an increased reaction time (i.e., divided attention, focused attention, flexibility, and integration of sensory information), an enhanced variability in reaction time (i.e., vigilance, divided attention, inhibition, focused attention, and flexibility) and a poorer task accuracy, as reported by an increased number of omission and/or commission errors (i.e., vigilance, divided attention, inhibition, focused attention, flexibility, and integration of sensory information) (39). With MPH treatment, patients with ADHD showed a significant improvement in accuracy in tasks related to vigilance, divided attention, inhibition, focused attention, flexibility, and integration of sensory information. The number of omission and commission errors in attention tasks was particularly sensitive to the medication (39).

Taken together, these studies underscore MPH positive effects on the attentional functioning of patients with ADHD (55, 56). However, children with ADHD on pharmacological therapy do not necessarily reach an undisturbed level of attentional functioning. Consequently, additional therapy should be considered since attention is a basic function for higher cognitive functioning, and persistent attention deficits are linked with poor social, academic, and occupational outcomes. Based on these considerations, Tucha and colleagues showed that an additional attention training programme led to significant improvements in various aspects of attention, including vigilance, divided attention and flexibility in children with ADHD who had received pharmacological treatment (55).

### Executive Functions and Their Neurobiological Bases

EFs refer to a set of mental control cognitive processes that permit the use of goal-directed behaviors and are represented by five main domains: response inhibition, planning, working memory, verbal fluency, and cognitive flexibility/shifting (57, 58). This last, but not least, domain may be interpreted as a sort of link between attentive and executive functioning. According to Barkley (59), EFs are those abilities that allow planning and organizing information in working memory and developing/evaluating an appropriate action from this information.

EFs have been conceptualized as a means of behavioral self-regulation that modulates behavior in a manner that is adaptive to the dynamic contexts of the situation at hand (60). Neuroanatomical and neurofunctional researches showed that the right inferior prefrontal cortex, the basal ganglia, and the subthalamic nucleus are crucial in controlling response inhibition (61, 62) and the anterior cingulate cortex (63), the lateral prefrontal cortex (63), and the parietal cortex (64) support the control of interference, a component of response inhibition. The dorsolateral prefrontal network, the anterior cingulate cortex, the orbitofrontal region, and the motor/premotor areas are involved in the processing necessary for planning and problem solving (65). Structural and functional neuroimaging research has supported the involvement of the prefrontal cortex (dorsolateral, ventrolateral, and rostral) and parietal cortex (bilateral and medial posterior) in correctly performing working memory tasks (66–69). The involvement of multiple frontal, cerebellar, temporal, and parietal regions has been described in association with both types of verbal fluency. Phonological fluency may be associated with the frontal regions, including the left motor/premotor, left or bilateral opercular, left lateral orbitofrontal, and right dorsolateral regions, whereas category fluency may be associated with temporal regions (70). Finally, cognitive flexibility is sub-served by the inferior parietal cortex, superior colliculus, posterior lateral thalamus, medial frontal areas, and pre-supplementary motor area (28).

### Executive Dysfunction and Methylphenidate

In addition to the three main clinical symptom dimensions (i.e., inattention, hyperactivity, and impulsiveness), children with ADHD also exhibit executive functioning deficits (57, 71), and in everyday life, they have problems in planning, organizing and finishing assigned tasks (29). Although executive functioning deficits are common in ADHD, they are not specific to the disorder. A meta-analysis of the literature in ADHD showed that response inhibition, working memory, and planning were the strongest and most consistent deficits found across studies (57). Difficulties in response inhibition (32, 72) and planning have been found in children with ADHD (73, 74). Working memory has been associated with short-term memory, and impairments in both domains have been reported in ADHD (75). Moreover, working memory has been consistently identified as problematic for patients with ADHD (32, 72), so much so, that the compromise of verbal and spatial working memory has been proposed as a possible neurocognitive trait of ADHD (32). The previous findings were confirmed by a recent study by Pitzianti and colleagues. Indeed, these authors found that compared with healthy peers, children with ADHD showed an impairment in measures of response inhibition, planning, and verbal working memory (76). In addition to the efficacy on main symptoms of ADHD, MPH also improves EF deficits in children suffering from this disorder (77–79).

It is known that the VNTR in the 3’UTR of DAT may influence the variability in the therapeutic response to MPH in individuals with ADHD (19, 80). In this framework, Pasini and colleagues (81) analyzed neuropsychological functioning after a prolonged period of MPH treatment and after a specific time of MPH suspension and the relationship between DAT VNTR genotypes and neurocognitive response to MPH in a sample of 108 drug-naïve children with ADHD. In this study, the performance of the enrolled subjects on measures of response inhibition, planning, and working memory was evaluated after 4, 8, and 24 weeks and at 8 weeks after MPH withdrawal. The patients with the 9/9 genotype showed an improvement in response inhibition and working memory only after 4 weeks of therapy and in planning after 24 weeks of treatment and after 8 weeks of MPH suspension. The children with the 9/10 genotype had an improvement in response inhibition after 4, 8, and 24 weeks of treatment and in planning after 24 weeks and after 8 weeks of MPH suspension. The children with the 10/10 genotype showed an improvement in response inhibition, planning, and working memory after 4, 8, and 24 weeks of treatment and after 8 weeks of suspension. Therefore, the 9/9 genotype seemed to be related to a worse response to MPH treatment; in contrast, the 10/10 genotype seemed to be related to a better response to MPH treatment (82). These results confirmed what was observed in previous researches on the association between DAT VNTR genotypes and response variability to MPH therapy (18, 19).

## Neurological Soft Signs and Methylphenidate

Neurological soft signs (NSSs) have been interpreted as minor neurological abnormalities in motor, sensory, and integrative functions (80). Some authors have suggested that NSS represent a failure in the integration between the sensory and motor system, whereas others have suggested deficits in neuronal circuits involving sub-cortical structures such as the basal ganglia and the limbic system (83). NSS are often observed in children with typical development and reflect the immaturity of the central nervous system, but their persistence into later childhood and adolescence suggested motor dysfunction and could be a marker of atypical neurodevelopment (84).

NSS are mainly represented by overflow movements (OMs) and dysrhythmia. OMs are defined as co-movements of body parts not specifically needed to efficiently complete a motor task (84). There are several different forms of OM: associated movements, contralateral motor irradiation, and mirror movements. Dysrhythmia is defined as an improper timing and/or rhythm of otherwise normal movement (85). OMs are related to a delay or defect of maturation in the intra-cortical and inter-cortical systems that support automatic inhibition (86), while dysrhythmia seems to be due to cerebellar dysfunction (87). In addition to the core symptoms, motor dysfunction is often present in patients with ADHD, and an increased number of OM (88), impaired timing of motor responses (89), deficits in motor coordination (90), and deficits in fine motor abilities (91) have frequently been reported in children with ADHD. Moreover, Meyer and Sagvolden found that children with ADHD performed worse on measures of manual dexterity, motor coordination, movement speed, accuracy, and stability of movement in comparison to healthy peers (92). These observations are in line with the results of subsequent studies that found multiple motor abnormalities, such as a greater number of OM, a greater dysrhythmia, and slowness in the speed of execution of timed movements in children with ADHD compared to healthy peers (93–95).

OMs likely reflect dysfunction within motor and premotor circuits that are important for the preparation and execution of motor responses (96). A functional neuroimaging research reported a smaller extent of activation in the contralateral primary motor cortex in patients with ADHD while performing a simple motor task. It may represent insufficient recruitment of the neuronal activity necessary to mobilize transcallosal interhemispheric inhibition (97). Dysfunctions in motor and premotor circuits, responsible for an increased prevalence rate of OM in patients with ADHD, may be due to abnormalities in white matter tracts, including the corpus callosum, which is important for the effective transfer of transcallosal inhibition (93–95). Dysrhythmia may represent cerebellar dysfunction (87), and the slowness of timed activities may be due to functional deficits in frontal-striatal networks, the cerebellum, and the basal ganglia structures (95). A study on the comorbidity between ADHD and high-functioning autism (HFA) revealed that children with ADHD had a greater number of NSS in comparison to healthy peers, patients with HFA showed a greater dysrhythmia and slowness compared to healthy children and patients with ADHD+HFA showed a greater number of NSS compared to healthy controls and a greater dysrhythmia compared to patients with ADHD. Therefore, through the NSS measure, it was possible to establish a gradient for the OM, in which children with ADHD were at one extreme (more OM) and children with HFA at the other extreme (less OM), while children with ADHD+HFA showed an OM number that was positioned in the middle between ADHD and HFA (41).

A study on the correlation between NSS and attentional dysfunction revealed that patients with ADHD were impaired in several attentional processes and reported a greater number of NSS compared to healthy children. Moreover, significant correlations between disturbances in attention and motor abnormalities were described in the clinical sample. Indeed, deficits in alertness (in terms of increased variability in reaction time) and sustained attention (in terms of an increase in reaction time and number of omission errors) interfered with speed-timed activities. Dysfunction of selective attention (in terms of increased number of omission errors) and divided attention (in terms of increased variability in reaction time in visual tasks and increased number of omission errors in auditory and visual tasks) significantly correlated with dysrhythmia. Impairments in sustained attention (in terms of increased number of omission errors) significantly correlated with OM. Taken together, these results suggested that attentional processes could be involved in the pathophysiology of NSS (76).

MPH treatment has been associated with the improvement or complete resolution of NSS and, in particular, of OM in patients with ADHD (98). It is known that OM may represent immaturity and/or dysfunction of the motor/premotor networks involved in motor response inhibition (97), which may be due to the volume reduction in these cerebral areas caused by the decrease in their white matter components, suggesting a primarily axonal abnormality in patients with ADHD (99).

The persistence of OM in patients with ADHD supports the hypothesis that the brain abnormalities in children suffering from this disorder have a developmental origin. Oligodendroglial abnormalities may be due to dysfunction of the DA system (100). MPH therapy causes an increase in DA signaling through multiple actions, including blockade of the DA reuptake transporter, amplification of DA response duration, disinhibition of D2r and amplification of DA tone (101). These effects could be relevant because D2r receptors are expressed during oligodendrocyte development and may also regulate the outgrowth of neuronal processes. The role played by MPH in regulating DA signaling during oligodendrocyte development could explain its positive effects on NSS and, in particular, on OM.

## Human Endogenous Retroviruses and Methylphenidate

Human endogenous retroviruses (HERVs) represent remnants from ancient germ line infections with exogenous retroviruses. During evolution, HERVs were amplified and spread throughout the human genome by repeated events of retrotransposition and/or reinfection, and they are integrated as provirus in chromosomal DNA (102). Their integration in any location of the human genome may alter the structure and/or function of genes (103). Although most HERV sequences are inactivated by mutations or deletions or silenced by epigenetic modifications (104), their potential responsiveness to environmental factors plays a relevant role in gene-environment interactions (105). Therefore, several lines of evidence have suggested that the inappropriate expression of HERV genes may initiate or maintain pathological processes and may be involved in the etiopathogenesis of many complex diseases, including neurological and psychiatric disorders, such as multiple sclerosis (106), schizophrenia, bipolar disorder (107), autism spectrum disorder (108, 109), and ADHD (110). Indeed, analyzing the expression levels of three HERV families (HERV-H, K, and W) in peripheral blood mononuclear cells (PBMCs) from patients with ADHD, Balestrieri and colleagues found the over-expression of HERV-H in subjects affected by this disorder in comparison to healthy controls, while there are no differences in the expression levels of HERV-K and W (110).

**Figure 2:**
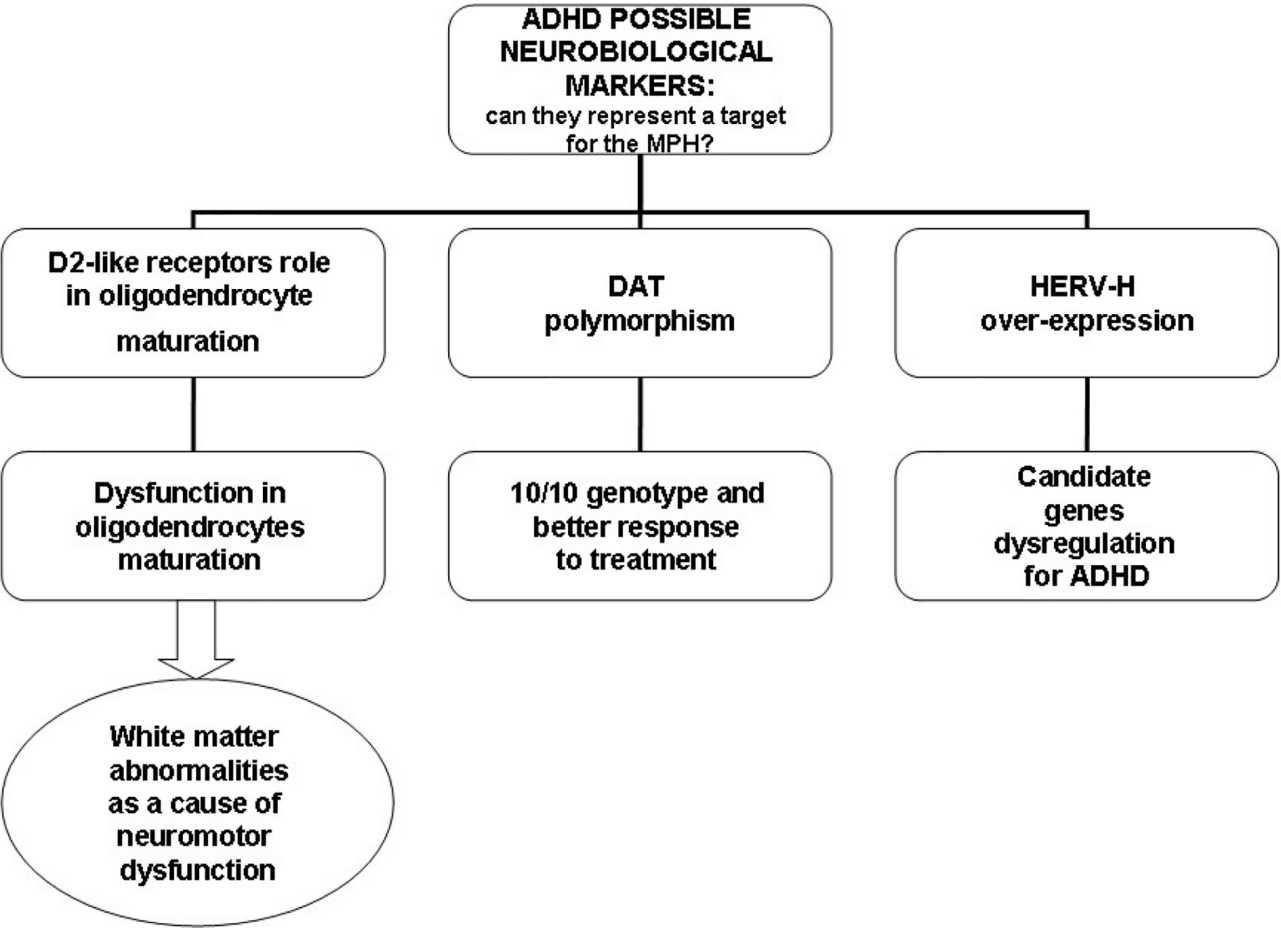
Possible neurobiological targets for methylphenidate (MPH).

In relation to the ability to integrate into the human genome and alter the structure and/or function of genes (102, 103), HERV-H could interfere with the functioning of candidate genes for ADHD, such as genes for DA, DA receptors, and DAT known to be involved in the etiopathogenesis of ADHD (1). Using real-time RT-PCR, D’Agati and colleagues investigated the influence of MPH on HERV transcription in PBMCs in a single case of a young patient with ADHD and described for the first time, a reduction in HERV-H expression and significant improvement in ADHD symptoms after 6 months of MPH treatment (111). This result was replicated in a larger sample of patients with ADHD. Balestrieri and colleagues found a fast reduction in HERV-H activity in patients with ADHD undergoing MPH therapy in parallel with an improvement in clinical symptoms (109, 110). Moreover, when PBMCs from drug-naïve patients were cultured *in vitro*, HERV-H expression increased, while no changes in the expression levels were found in ADHD patients undergoing therapy. This suggests that MPH could affect HERV-H activity (112). **Figure 2** illustrates possible neurobiological targets for MPH.

## Conclusions

Therapy with MPH in children with ADHD is accompanied by improvements in attentional and executive dysfunction. Although MPH-induced improvements are reported across a broad range of attention measures, children with ADHD do not necessarily reach an undisturbed level of attentional functioning. Consequently, additional treatment based on attention training programmes would be desirable. However, marked improvement or complete resolution of NSS following treatment with MPH in patients with ADHD was described. At this regard, the evaluation of NSS, as a marker of atypical neurodevelopment, may be useful for monitoring the effectiveness of pharmacological treatment with MPH in children with ADHD.

Scientific evidence has suggested that the DAT polymorphisms could be considered a predictor of the MPH therapeutic response, pointing to targets for a precision medicine approach. It is interesting that patients with ADHD who do not respond to MPH have a low primary striatal DAT availability, whereas patients with a better response to MPH treatment have a higher DAT concentration. Recent studies showed higher striatal DAT concentrations in subjects homozygous for the 10/10 genotype. It has been reported that MPH lowers DAT striatal availability very effectively in patients with ADHD, and this coincides with clinical improvement.

Finally, although further studies are needed at this regard, the most advanced studies have suggested that HERVs play a role in the etiopathogenesis of ADHD and HERV-H over-expression could be considered a biologically distinct trait of patients with ADHD. MPH administration has been associated with a reduction in HERV-H activity that coincides with improvement in the core symptoms of the disorder.

## Author Contributions

MP wrote the first draft of the manuscript and was in charge of the patient’s management. SS and EB performed the literature analysis. SE critically revised the text and made substantial scientific contributions. AP co-wrote the first draft of the manuscript. All authors contributed to the article and approved the submitted version.

## Funding

This review has been supported by Autopilot DX Grant (Pediatric Section, University of Perugia, Perugia, Italy).

## Conflict of Interest

The authors declare that the research was conducted in the absence of any commercial or financial relationships that could be construed as a potential conflict of interest.
